# Complete Genome Sequence of *Staphylococcus aureus* Phage GRCS

**DOI:** 10.1128/genomeA.00209-14

**Published:** 2014-04-10

**Authors:** Steven M. Swift, Daniel C. Nelson

**Affiliations:** a Institute for Bioscience and Biotechnology Research, University of Maryland, Rockville, Maryland, USA; b Department of Veterinary Medicine, University of Maryland, College Park, Maryland, USA

## Abstract

The *Staphylococcus aureus* phage GRCS was isolated from a sewage treatment facility in India and has shown potential for phage therapy in a mouse model of bacteremia. Here, we report the complete genome sequence of this bacteriophage.

## GENOME ANNOUNCEMENT

The bacteriophage GRCS was isolated from raw sewage collected from a treatment plant in India (1). GRCS is a lytic phage that infects *Staphylococcus aureus* and has been shown to protect mice against bacteremia caused by *S. aureus*. This bacteria is a common cause of infections in humans, and drug-resistant strains are becoming more common (2). Therefore, the GRCS phage has potential for phage therapy to treat *S. aureus* infections.

Genomic DNA was isolated from GRCS phage amplified in *S. aureus* strain NRS382. GRCS has a linear double-stranded DNA genome. Genomic DNA was digested with various restriction enzymes (MfeI, BsrGI, HindIII, SspI, and AccI) and cloned into plasmids pUC19 and pJET1-2. The clones were sequenced with plasmid-derived primers. The sequences from the cloned genomic fragments were used to design primers for direct sequencing of the genomic DNA. Sanger sequencing was done with ABI 3730xl sequencers, and base calls were made using Phred (3) at Macrogen USA (http://www.macrogenusa.net/). The sequence reads were trimmed of vector and poor-quality sequences using the preGap4 (version 1.6) and Trev (version 1.9) programs, and then they were assembled using the Gap4 program (version 4.11.2) of the Staden package (http://staden.sourceforge.net/) (4). The genome was assembled from 97 sequence reads, with an average read length of 932 bases, into a single 17,869-base contig. There are an average of 5 sequence reads per base in the consensus sequence.

Analysis of the GRCS genome by the FgenesB program (5) found 21 genes. The G+C content of the phage genome is 28.90%. No tRNA genes were detected by the tRNAscan-SE program (6). The protein functions of the 21 genes were determined by protein BLAST (7) and by CD-Search (8). Ten of the predicted genes are conserved hypothetical proteins. The phage genes with predicted protein functions include those for holin, endolysin, major and minor tail proteins, upper and lower collar proteins, major capsid protein, single-stranded DNA binding protein, and DNA polymerase.

Electron microscope images from our lab indicate that morphologically, GRCS belongs to the *Podoviridae* family due to the presence of short noncontractile tails (D. C. Nelson, unpublished data). By nucleotide BLAST search of the genome, the nearest neighbors (identity range, 89 to 91%) of GRCS are *Staphylococcus* phages phi44AHJD, phage 66, phiP68, SAP-2, S13′, and S24-1. Of these phages, the first four are classified by the International Committee on the Taxonomy of Viruses (ICTV) and the National Center for Biotechnology Information (NCBI) taxonomy browser as belonging to the *Podoviridae* family, *Picovirinae* subfamily, and *Ahjdlikevirus* genus. S13′ and S24-1 are listed as unclassified members of the *Picovirinae* subfamily. Thus, GRCS is a member of a small group of closely related *S. aureus* phages.

### Nucleotide sequence accession number.

The complete genome of bacteriophage GRCS has been deposited in GenBank under the accession no. KJ210330. The version described in this paper is the first version.
